# Supplementary material for: Moisture-tolerant Mg-metal electrodes for practical fabrication of rechargeable Mg batteries
Source: Nat Commun. 2026 Mar 9;17:3678. doi: 10.1038/s41467-026-70378-3 (PMC13100027; doi:10.1038/s41467-026-70378-3)
Supplement: Supplementary file 1 — Supplementary Information [file 41467_2026_70378_MOESM1_ESM.pdf]

Supplementary Information for

Moisture-tolerant Mg-metal electrodes for practical fabrication of rechargeable Mg batteries

Woo Joo No[1,2†], Jonghyun Han[1†], Jinyeon Hwang[1†], Sibylle Riedel[3], Minji Jeong[1], Hyeong Kyu Park[4], Ju Young Kim[2,5], Kwan Young Lee[2], Minah Lee[1], Taeeun Yim[6], Hyun Deog Yoo[7], Hyung Chul Ham[4], Sang-Young Lee[8], Zhirong Zhao-Karger[3,9], and Si Hyoung Oh[1,10]*

[1] Energy Storage Research Center, Korea Institute of Science Technology (KIST); Hwarang-ro 14-gil 5, Seongbuk-gu, Seoul 02792, Republic of Korea

[2] Department of Chemical and Biological Engineering, Korea University; 145, Anam-ro, Seongbuk-gu, Seoul 02841, Republic of Korea

[3] Helmholtz Institute Ulm (HIU), Electrochemical Storage; Helmholtzstrasse 11, 89081 Ulm, Germany

[4] Department of Chemical Engineering, Inha University; Inha-ro 100, Michuhol-gu, Incheon 22212, Republic of Korea

[5] Clean Energy Research Center, Korea Institute of Science Technology; Hwarang-ro 14-gil 5, Seongbuk-gu, Seoul 02792, Republic of Korea

[6] Department of Chemistry, Incheon National University; 119 Academy-ro, Yeonsu-gu, Incheon 22012, Republic of Korea

[7] Department of Chemistry and Chemical Institute for Functional Materials, Pusan National University; Busan 46241, Republic of Korea

[8] Department of Chemical and Biomolecular Engineering, Yonsei University; 262 Seongsanno, Seodaemun-gu, Seoul 03277, Korea.

[9] Institute of Nanotechnology (INT), Karlsruhe Institute of Technology (KIT); Hermann-von-Helmholtz Platz 1, 76344 Eggenstein-Leopoldshafen, Germany

[10] Division of Energy & Environment Technology, Korea University of Science and Technology; Hwarang-ro 14-gil 5, Seongbuk-gu, Seoul 02792, Republic of Korea

[*]Corresponding author. Email: sho74@kist.re.kr

[†]These authors contributed equally to this work.

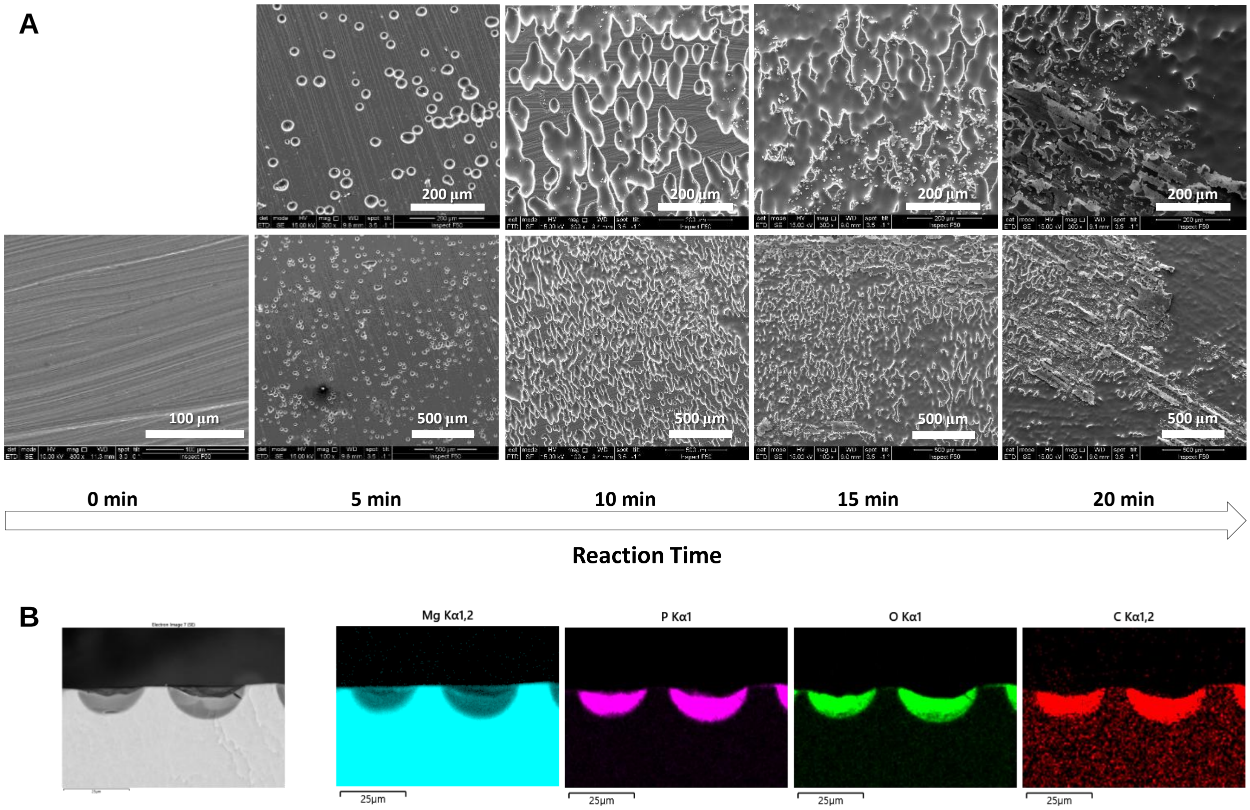

Supplementary Fig. 1. (A) Morphological evolution of magnesium surface over reaction time upon dipping Mg foil in trimethyl phosphate (TMP). The optimal treatment time was determined to be 15 min., when most surface area was reacted, being covered with smooth surface film. Excessive reaction time leads to blackening, pulverization and peeling-off of reaction products. (B) The cross-sectional image of TMP-treated Mg metal for 15 min. The elemental analysis indicate that the surface is covered with phosphate-based reaction products.

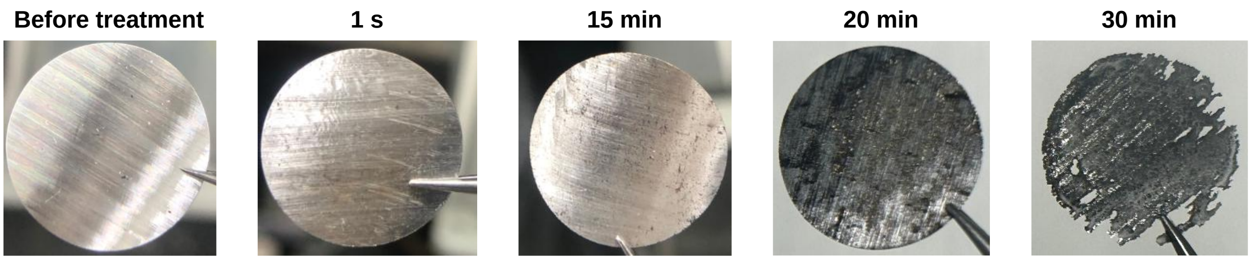

Supplementary Fig. 2. Photographs of Mg metal foil disks before and after trimethyl phosphate (TMP) treatment (after 1 seconds and after up to 30 minutes). The initial metallic luster began to wear off as reaction time went by. After 1 day of dipping in TMP, Mg metal was completely dissolved.

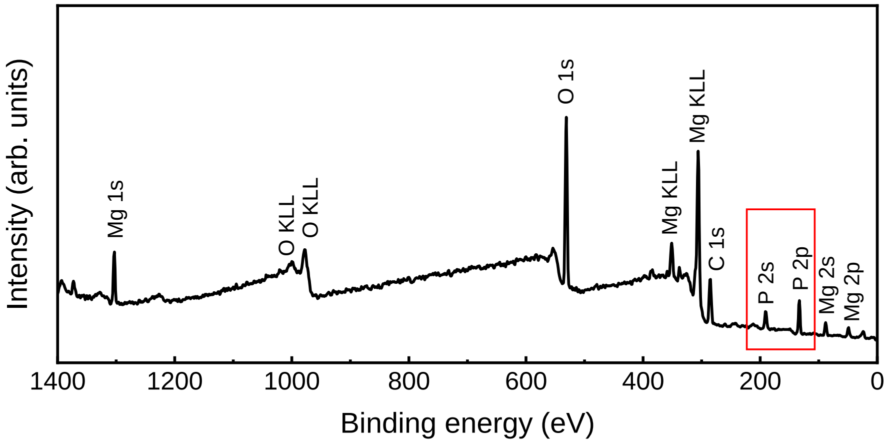

	Atom. %
C 1s	16.4
O 1s	44.0
Mg 1s	28.9
Mg 2p	10.8
P 2p	0.1

Supplementary Fig. 3. X-ray photoelectron spectroscopy spectra (survey scan) of trimethyl phosphate (TMP)-treated Mg electrodes and corresponding elemental analysis. The distinct P 2*s* and P 2*p* spectra indicate the existence of phosphate-based reaction products on the surface.

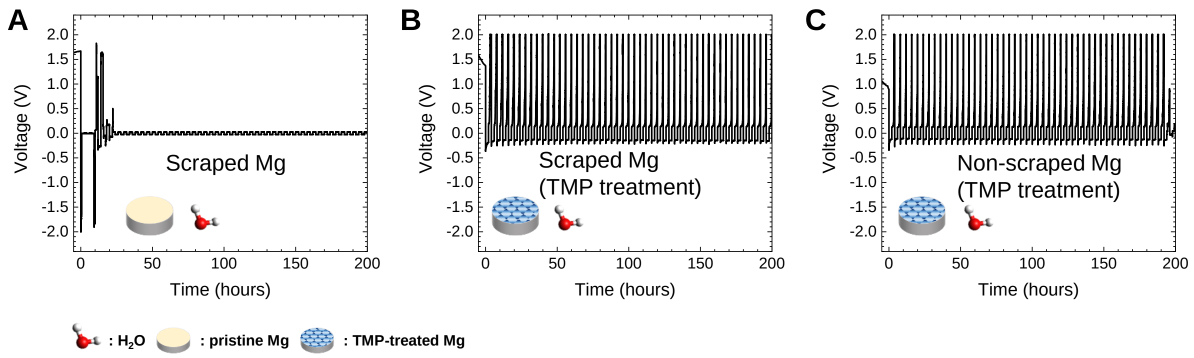

Supplementary Fig. 4. Electrochemical characterization of trimethyl phosphate (TMP)-treated Mg electrodes in moist (314 ppm H_2O) E2 electrolyte (0.5M $Mg(TFSI)_2$ + 0.5 M $MgCl_2$ in G2). Mg plating-stripping behavior with of Mg||SS asymmetric cells composed (A) of scraped untreated Mg electrode, and a stainless steel foil working electrode, (B) of TMP-treated Mg electrode, and a stainless steel foil working electrode, and (C) of TMP-treated Mg(non-scraped) electrode, and a stainless steel foil working electrode. TMP-treated Mg(non-scraped) electrode was prepared by reacting TMP with a Mg foil disk for one hour whose native oxide was not removed beforehand. In contrast to scraped Mg, it took at least one hour to activate non-scraped Mg electrode with TMP, as evidenced by the gradual increase in the number and size of reaction pits over time (Supplementary Fig. 38). The reaction between TMP and non-scraped Mg proceeded slowly, likely because the native oxide layer was almost insoluble in TMP (Supplementary Table 4) and it took considerable time for TMP molecules to penetrate to the bare Mg surface along the grain boundary or defect sites on the surface.

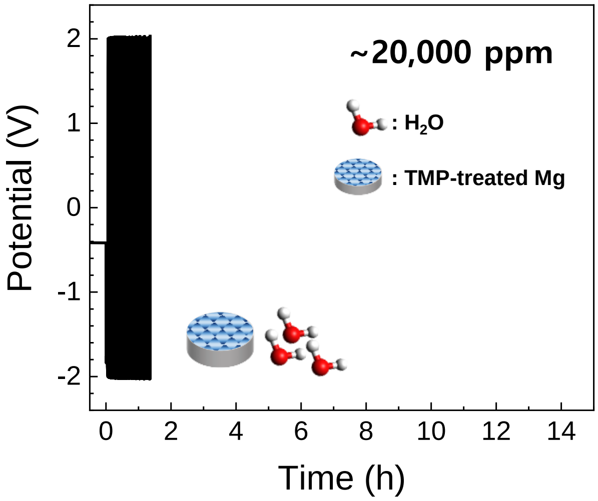

Supplementary Fig. 5. Mg plating-stripping behavior of a Mg||Mg symmetric cell with a pair of trimethyl phosphate (TMP)-treated Mg electrodes in moist E1 electrolyte containing 20000 ppm H_2O. The current density applied was fixed at 0.1 mA cm^{-2}. An excessive overpotential development indicated that there was a limit in the moisture tolerance in TMP-treated Mg electrode. However, the protected electrode can maintain the reversibility with the water content of at least thousands of ppm in the electrolytes, which would suffice to endure the moisture uptake from the atmosphere during the battery manufacturing process in a typical dry room facility.

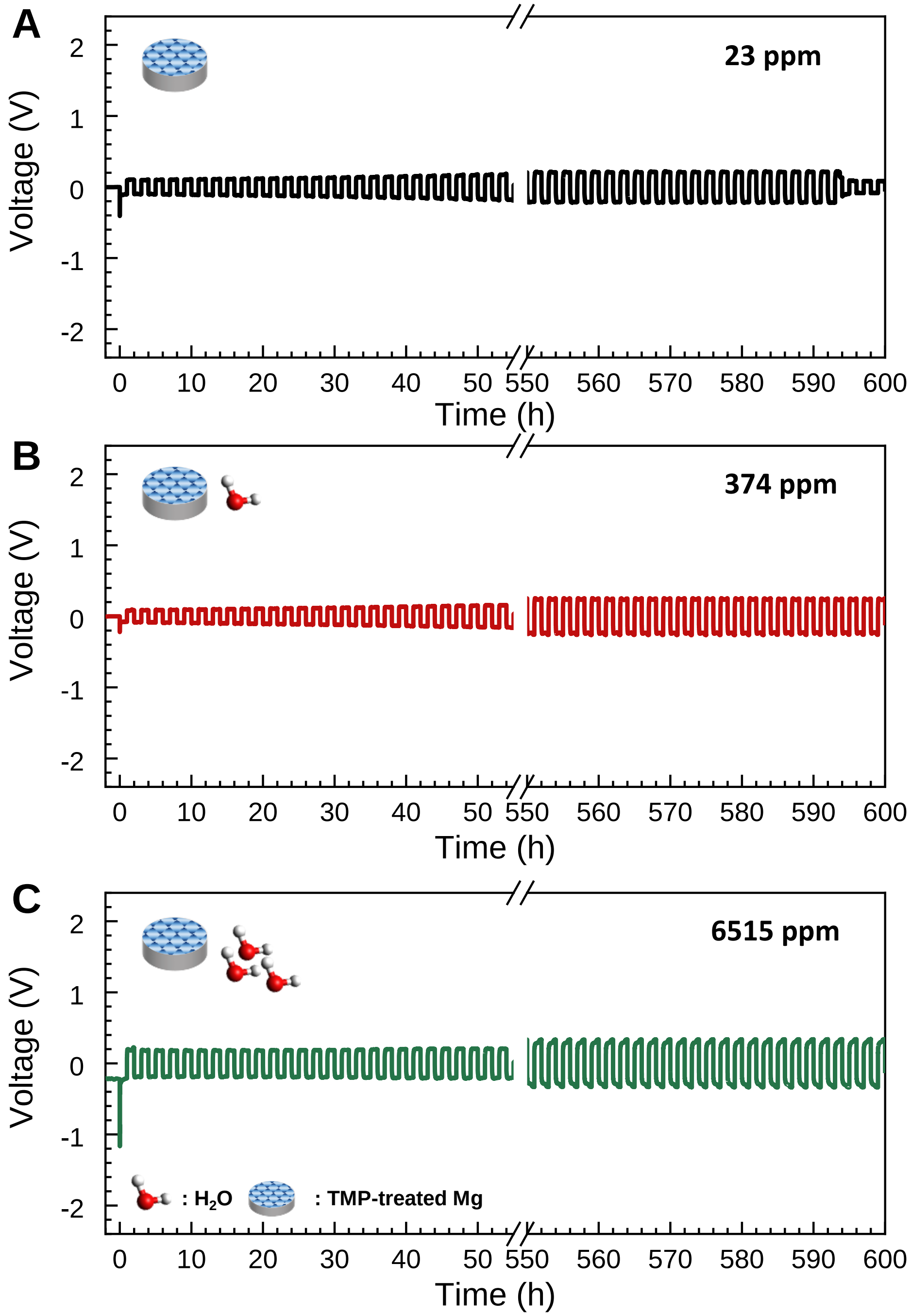

Supplementary Fig. 6. The voltage profiles during the prolonged Mg plating-stripping cycling of Mg||Mg symmetric cells made of a pair of trimethyl phosphate (TMP)-treated Mg electrodes in E1 electrolytes having various moisture contents, (A) 23, (B) 374, and (C) 6515 ppm H_2O. The current density applied and deposition amount of Mg were fixed at 0.1 mA cm^{-2}, and 0.1 mAh cm^{-2}, respectively. In all cases, reversible Mg plating-stripping was distinctly observed.

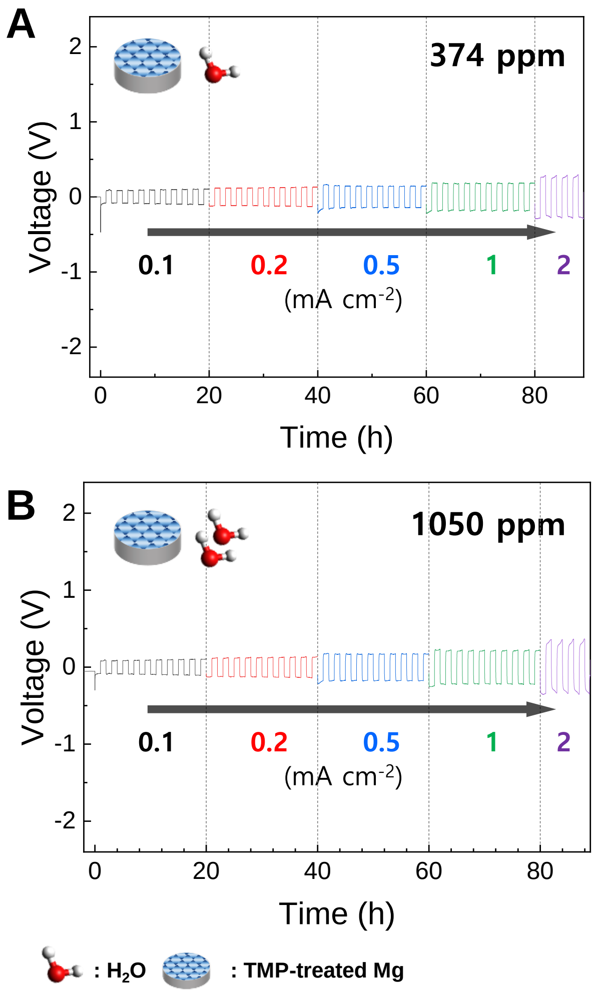

Supplementary Fig. 7. Mg plating-stripping behavior under various current rates (0.1, 0.2, 0.5, 1, 2 mA cm^{-2}) in the El electrolytes with (A) medium- (374 ppm H_2O) and (B) high- (1050 ppm H_2O) level of moisture contents. The deposition was carried out for 1 h for each current rate.

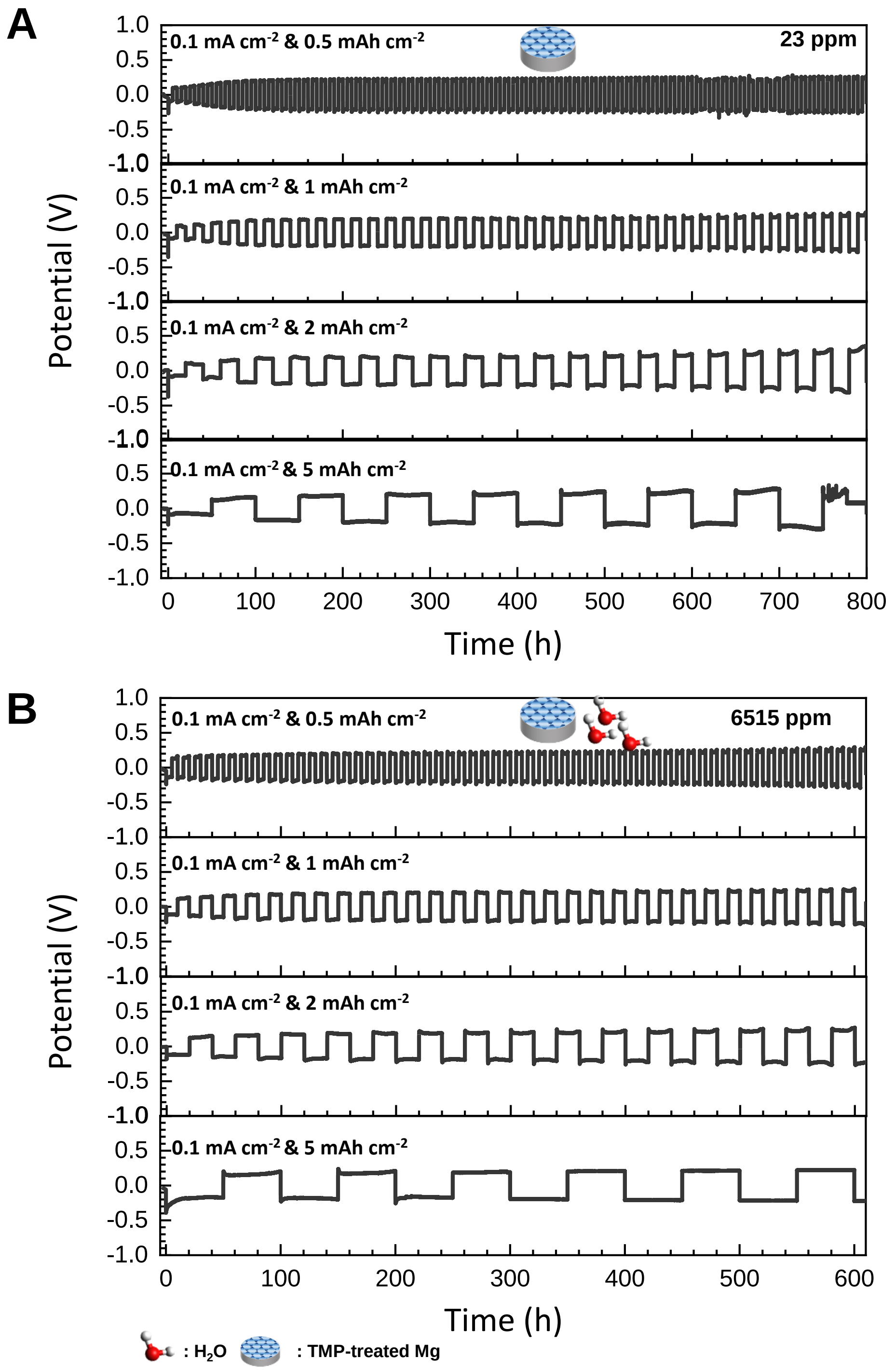

Supplementary Fig. 8. Mg plating-stripping behavior under various Mg deposition quantities (0.5, 1, 2, 5 mAh cm^{-2}) in E1 electrolyte at (A) dry (23 ppm H_2O) and (B) highly moist (6,515 ppm H_2O) conditions. The current density applied was fixed at 0.1 mA cm^{-2}.

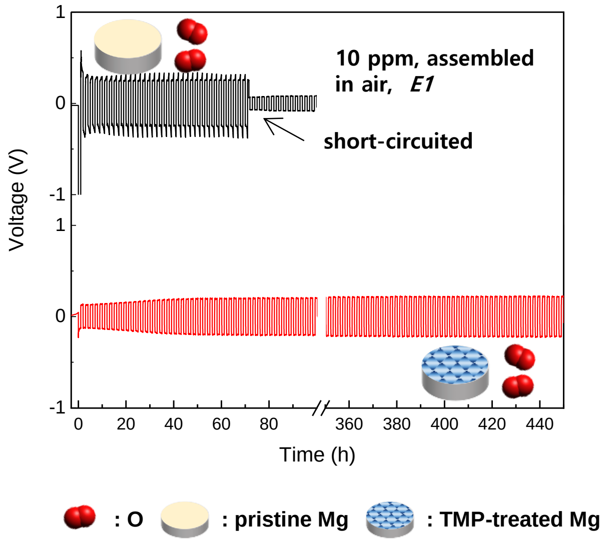

Supplementary Fig. 9. Mg plating-stripping behaviors of a cell comprised of a pair of untreated Mg electrodes and a cell consisting of a pair of trimethyl phosphate (TMP)-treated Mg electrodes in the dry electrolyte (10 ppm H_2O) assembled under atmospheric condition in a dry-room. The cell composed of a pair of untreated Mg electrodes worked normally at the beginning, but short-circuited soon after 70 h of operation, while the cell composed of a pair of TMP-treated Mg electrodes exhibited stable potential profiles for hundreds of cycles.

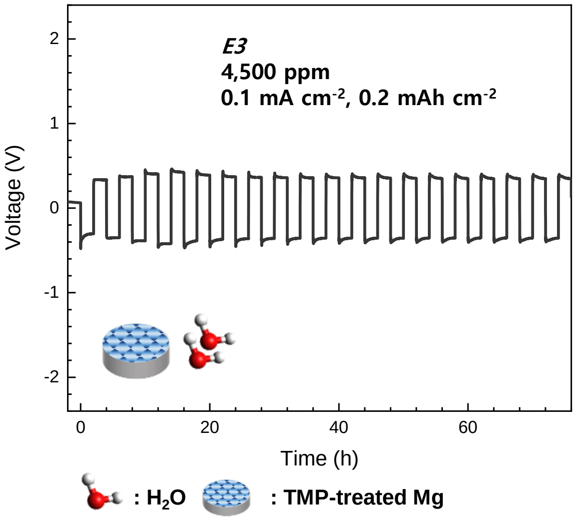

Supplementary Fig. 10. Mg plating-stripping behavior of a cell made of a pair of trimethyl phosphate (TMP)-treated Mg electrodes under highly moist E3 electrolyte containing 4500 ppm H_2O. The plating-stripping was carried out in 0.1 mA cm^{-2}, 0.2 mAh cm^{-2}.

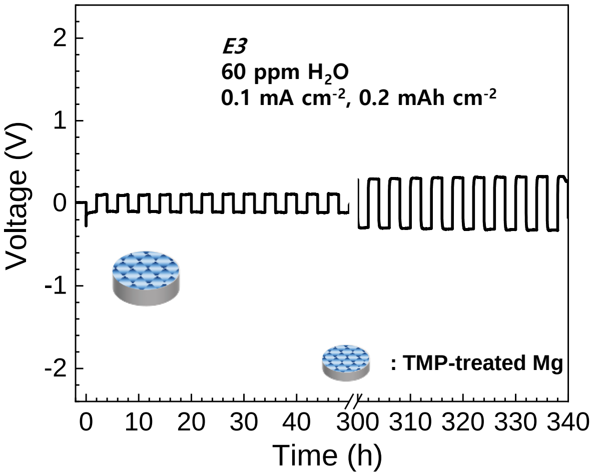

Supplementary Fig. 11. The voltage profile evolutions during the extended Mg plating-stripping cycling (~350 h) of Mg||Mg symmetric cell composed of a pair of trimethyl phosphate (TMP)-treated Mg electrodes in a chloride-free E3 electrolyte containing 60 ppm H_2O. The plating-stripping was carried out in 0.1 mA cm^{-2}, 0.2 mAh cm^{-2}.

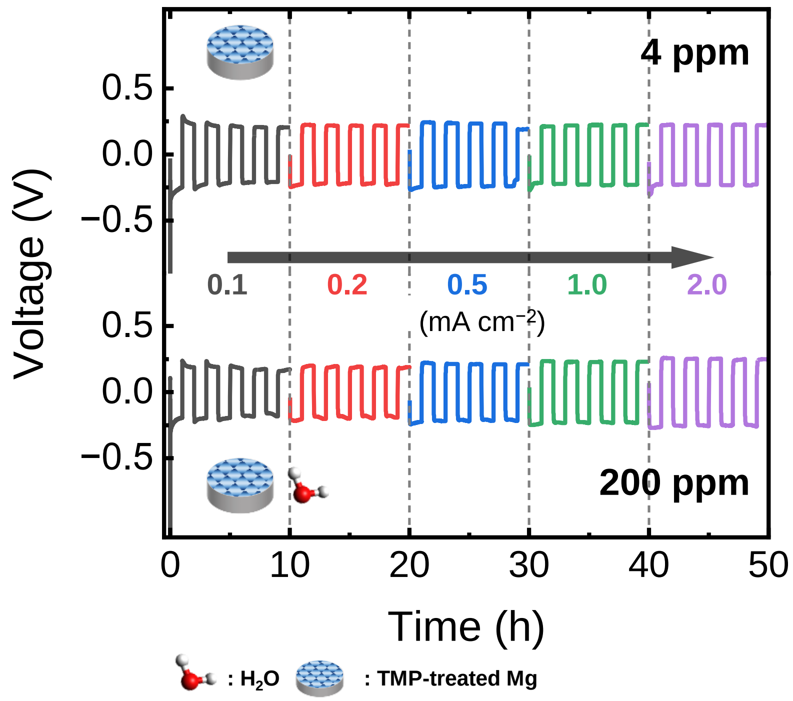

Supplementary Fig. 12. Mg plating-stripping behavior under various current rates (0.1, 0.2, 0.5, 1, 2 mA cm^{-2}) in the E3 electrolytes with 4 and 200 ppm H_2O. The deposition was carried out for 1 h for each current rate.

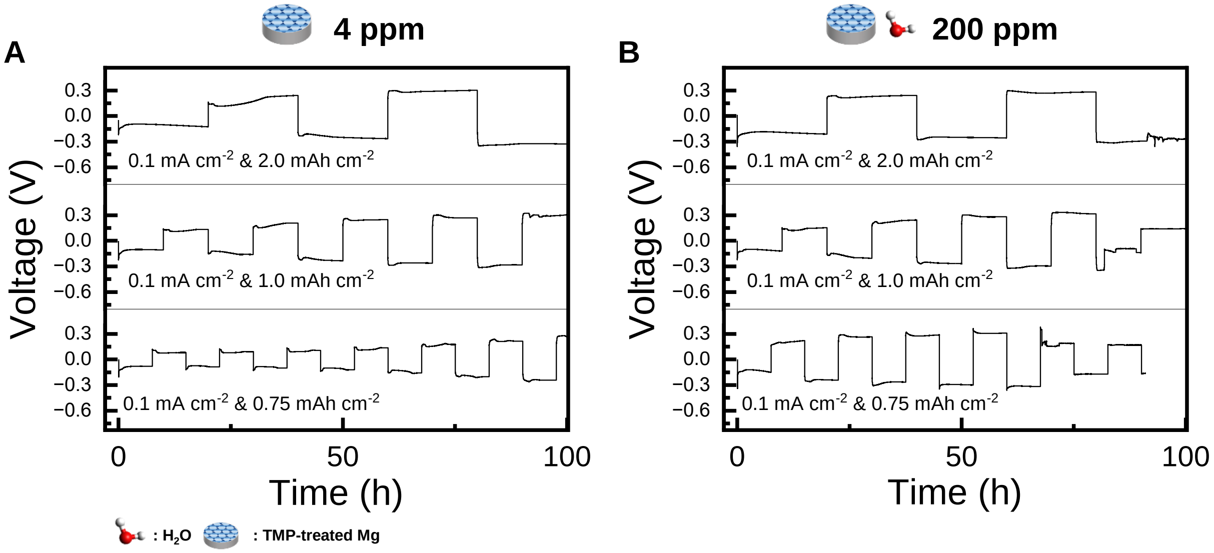

Supplementary Fig. 13. Mg plating-stripping behavior under various Mg areal capacities (0.75, 1, 2 mAh cm^{-2}) in E3 electrolyte at (A) dry (4 ppm H_2O) and (B) moist (200 ppm H_2O) conditions. The current density applied was fixed at 0.1 mA cm^{-2}.

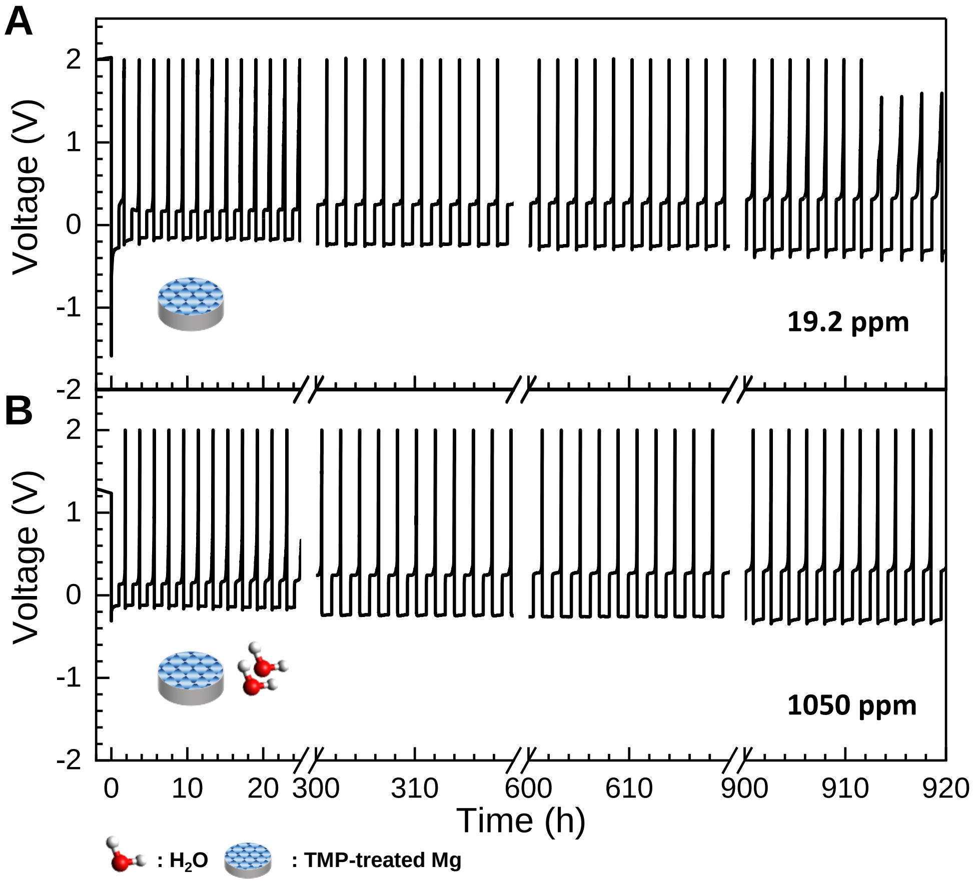

Supplementary Fig. 14. Electrochemical characterization of trimethyl phosphate (TMP)-treated Mg electrode in dry and moist E1 electrolyte with Mg||SS asymmetric configuration. Mg plating-stripping behavior in (A) dry (19.2 ppm H_2O), and (B) moist (1050 ppm H_2O) electrolytes. The plating-stripping was carried out in 0.1 mA cm^{-2}, and 0.1 mAh cm^{-2}. With TMP-treated Mg electrode applied, Mg plating-stripping was reversible regardless of moisture level in the electrolyte even with an asymmetric configuration. This indicates that the moisture content in the electrolyte is quickly removed by some scavenging mechanism provided by TMP-treated Mg electrode.

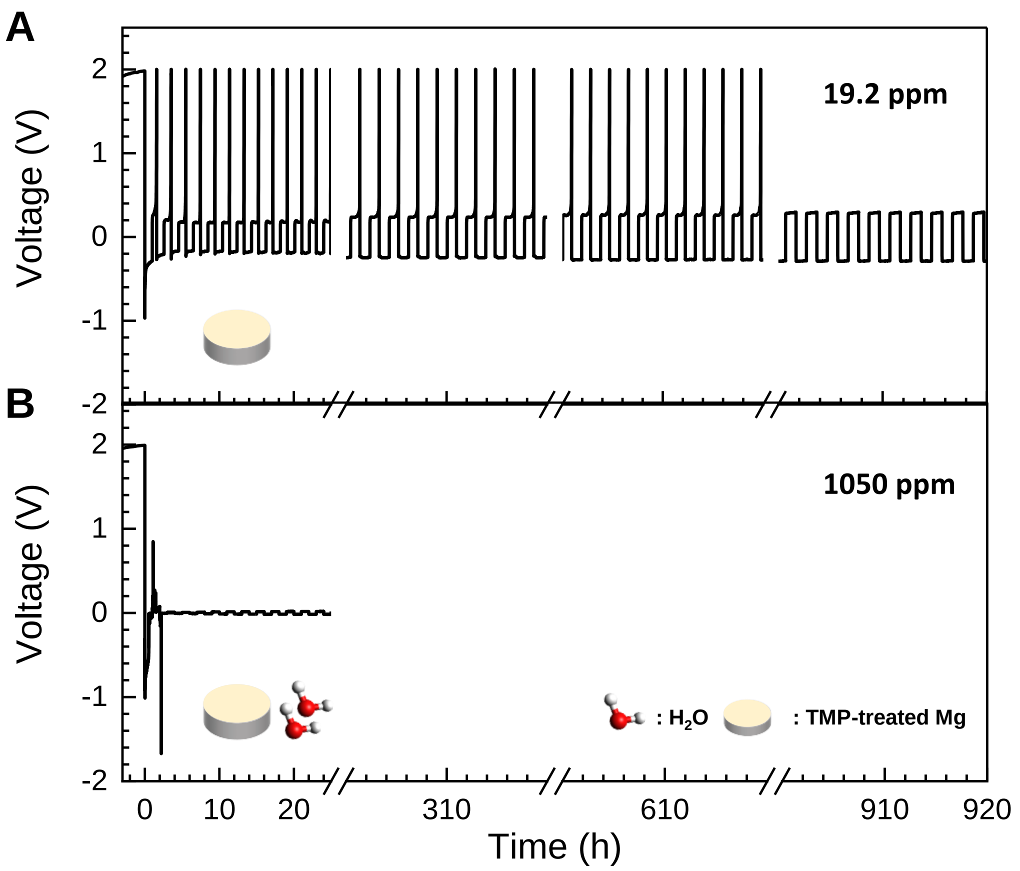

Supplementary Fig. 15. Electrochemical characterization of the scraped untreated Mg electrodes in dry and moist E1 electrolyte with Mg||SS asymmetric configuration. Mg plating-stripping behavior was shown in (A) dry (19.2 ppm H_2O), and (B) moist (1,050 ppm H_2O) electrolytes. In the moist electrolyte, Mg plating-stripping is highly irreversible with an untreated Mg electrode as opposed to trimethyl phosphate (TMP)-treated electrode (Supplementary Fig. 14).

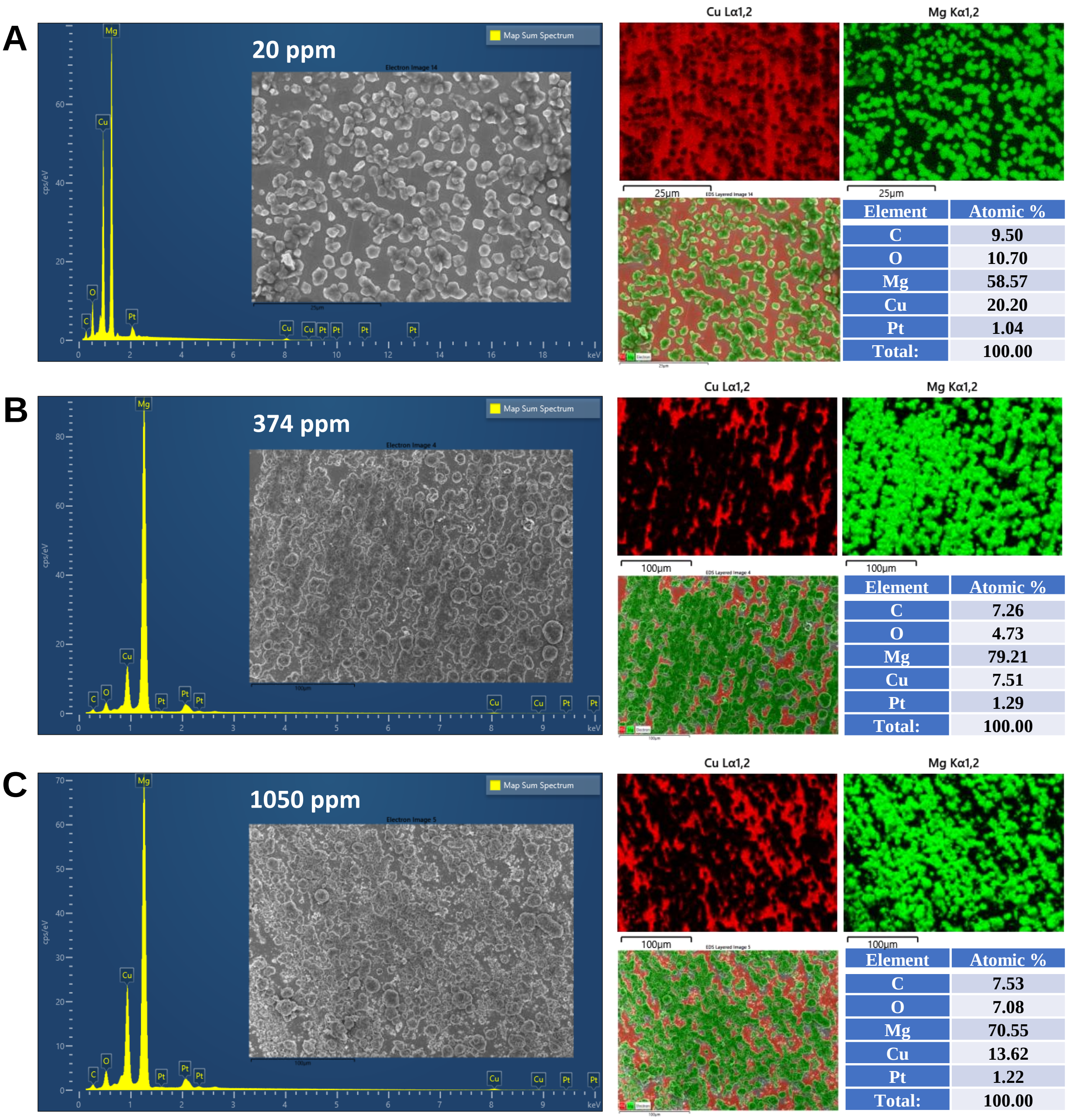

Supplementary Fig. 16. Morphological and elemental analyses on Mg deposits plated on Cu foil from the E1 electrolytes with various moisture level, e.g., (A) 20 ppm H_2O, (B) 374 ppm H_2O, and (C) 1,050 ppm H_2O. The plating was carried out in Mg||Cu asymmetric cells with 0.1 mA cm^{-2}, and 0.1 mAh cm^{-2}. Mg deposits were typically shaped as round spheroids regardless of moisture content in the electrolyte. This may be caused by quick moisture scavenging mechanism provided by trimethyl phosphate (TMP)-treated Mg electrode.

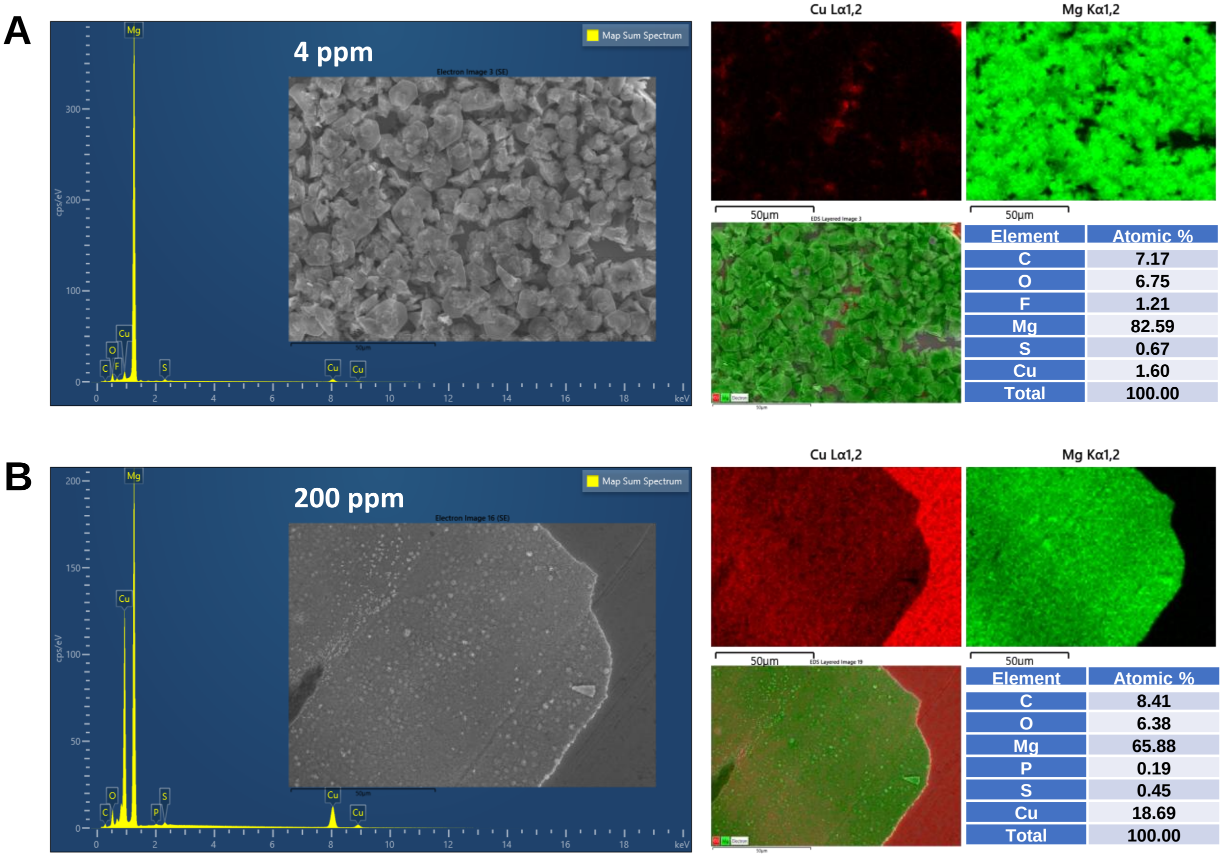

Supplementary Fig. 17. Morphological and elemental analyses on Mg deposits plated on Cu foil from the E3 electrolytes with various moisture level, e.g., (A) 4 ppm H_2O, and (B) 200 ppm H_2O. The plating was carried out in Mg||Cu asymmetric cells with 0.1 mA cm^{-2}, and 0.1 mAh cm^{-2}.

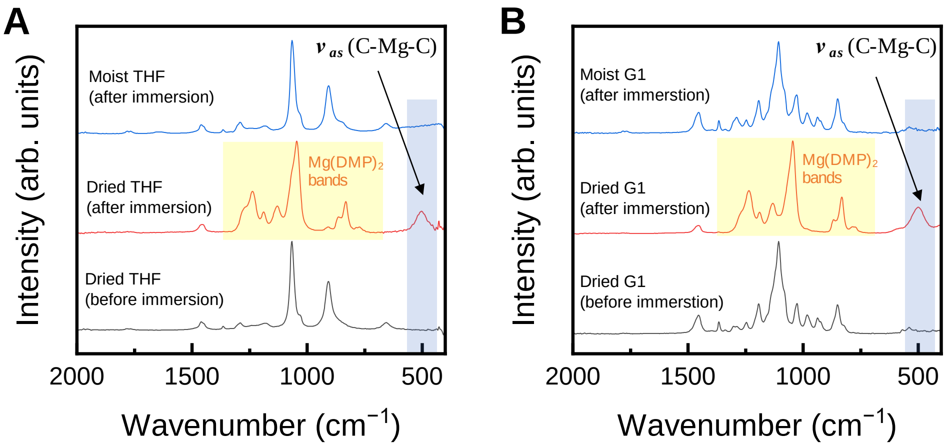

Supplementary Fig. 18. Fourier transform–Infrared (FT-IR) spectra of the (A) THF and (B) G1 solutions before and after immersion of a piece of trimethyl phosphate (TMP)-treated Mg disk for 10 min. Each solvent was prepared in a highly dried (< 10 ppm H_2O) and moist (~6500 ppm H_2O) conditions. The absorption peak at ~500 cm^{-1} could be assigned to asymmetric stretching mode of C-Mg-C bond of Me_2Mg as reported.[1] Therefore, after immersion in the solvent, Me_2Mg released from the TMP-treated Mg is expected to remain dissolved in the dried solvent, whereas it is unlikely to survive in a highly moist solvent. Most of the peaks beyond 500 cm^{-1} in the FT-IR spectra of dry solvents exposed to the treated Mg electrodes correspond to the characteristic bands of $Mg(DMP)_2$, as confirmed in Supplementary Fig. 19. $Mg(DMP)_2$ exhibits partial solubility in highly dried (<10 ppm H_2O) DME and THF, which explains the appearance of these peaks in the spectra of the solvent phase. In contrast, when water is present (in moist electrolyte), $Mg(DMP)_2$ becomes insoluble. As shown in Supplementary Fig. 20, the TMP-treated Mg electrodes immersed in moist G1 or THF display distinct $Mg(DMP)_2$ peaks, whereas those immersed in highly dried G1 or THF show much weaker features. This indicates that once $Mg(DMP)_2$ adsorbs water molecules from the moist electrolytes, it precipitates onto the electrode the surface, reducing its concentration in the bulk solution. Such behavior is consistent with the hygroscopic nature of $Mg(DMP)_2$, and its decreased solubility in ethereal solutions upon exposure to moisture. To confirm this, 1 mL of moist electrolyte was added to 1 mL of highly dried solvent that had been previously exposed to the treated Mg and examined the resulting FT-IR spectra (Supplementary Fig. 21). The peaks associated with $Mg(DMP)_2$ disappeared completely, and the spectra reverted to those of the pure solvents or to those observed when the TMP-treated Mg was directly immersed in the moist solvents. This demonstrates that $Mg(DMP)_2$ species dissolved

in the dry solvents react with the added water, becoming insoluble and precipitating from the solution, as also observed on the electrode surface.

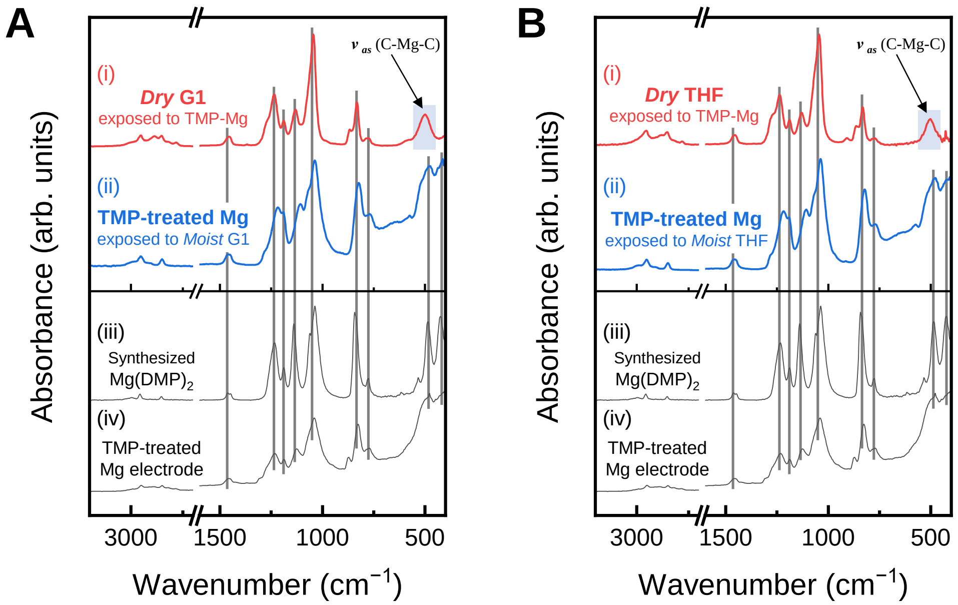

Supplementary Fig. 19. Fourier transform–infrared (FT-IR) spectra of (i) the highly dried solvent (<10 ppm H_2O) exposed to trimethyl phosphate (TMP)-treated Mg, and (ii) TMP-treated Mg electrode exposed to moist solvent (~8000 ppm H_2O). The solvent for (A) is G1, and that for (B) is THF. The red spectra, (i), represent the absorbance of dried solution after immersion of TMP-treated Mg. The blue spectra, (ii), correspond to the TMP-treated Mg exposed to moist solution. Synthesized $Mg(DMP)_2$, (iii), and TMP-treated Mg electrode, (iv), samples are shown as references. The volume of all solutions was 1 mL, and the analysis was performed after a 15 min reaction time.

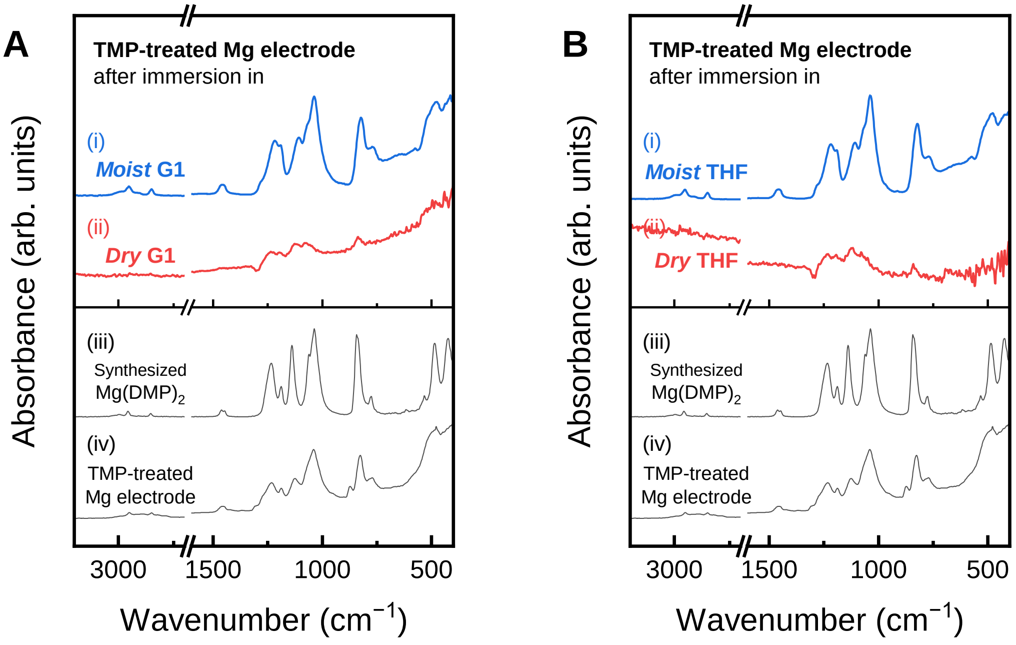

Supplementary Fig. 20. Fourier transform–infrared (FT-IR) spectra of trimethyl phosphate (TMP)-treated Mg electrodes after immersion in with (A) G1 and (B) THF. Each solution was prepared in a highly dried (< 10 ppm H_2O) and moist (~8000 ppm H_2O) conditions. The blue, (i), and the red, (ii), spectra represent the absorbance of TMP-treated Mg electrodes after immersion in moist and dried solvent, respectively. Synthesized $Mg(DMP)_2$ (iii) and TMP-treated Mg samples (iv) are shown as references. The volume of all solutions was 1 mL, and the analysis was performed after a 15 min reaction time.

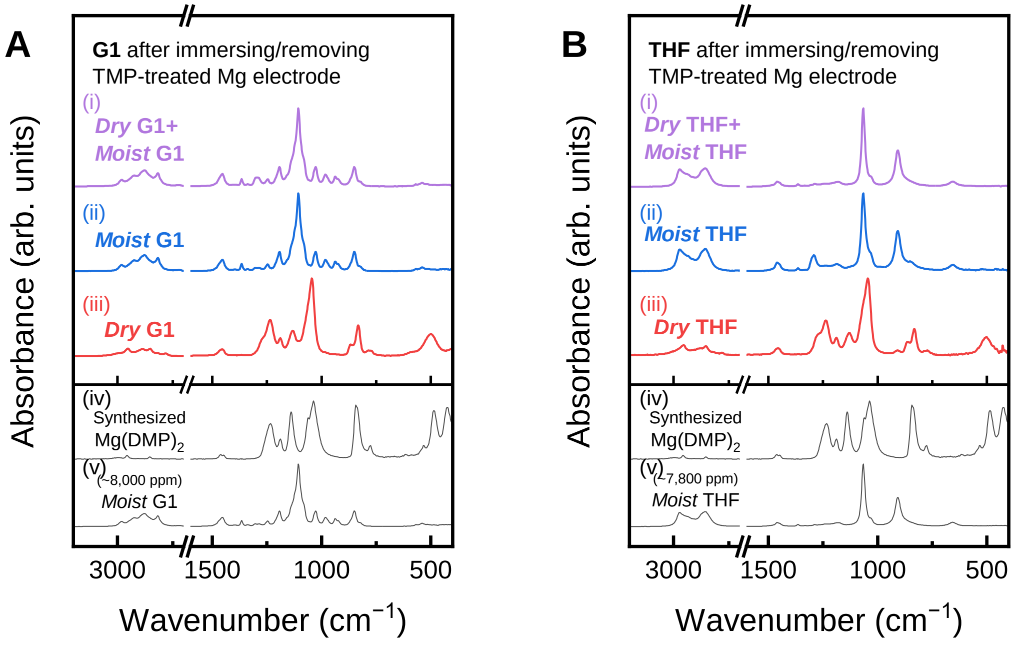

Supplementary Fig. 21. Fourier transform–infrared (FT-IR) spectra of (A) G1 and (B) THF after immersing / removing trimethyl phosphate (TMP)-treated Mg electrodes. The purple, (i), spectra correspond to the case 1 mL of moist electrolyte (~8000 ppm H_2O) was added to 1 mL of highly dried solvent (<10 ppm) that had been previously exposed to treated Mg. The blue, (ii), and red, (iii), spectra represent the absorbance of moist and dried solvent after immersion of TMP-treated Mg, respectively. Synthesized $Mg(DMP)_2$, (iv), and TMP-treated Mg samples, (v), are shown as references. The volume for (ii), and (iii) was 1 mL, and the analysis was performed after a 15 min reaction time.

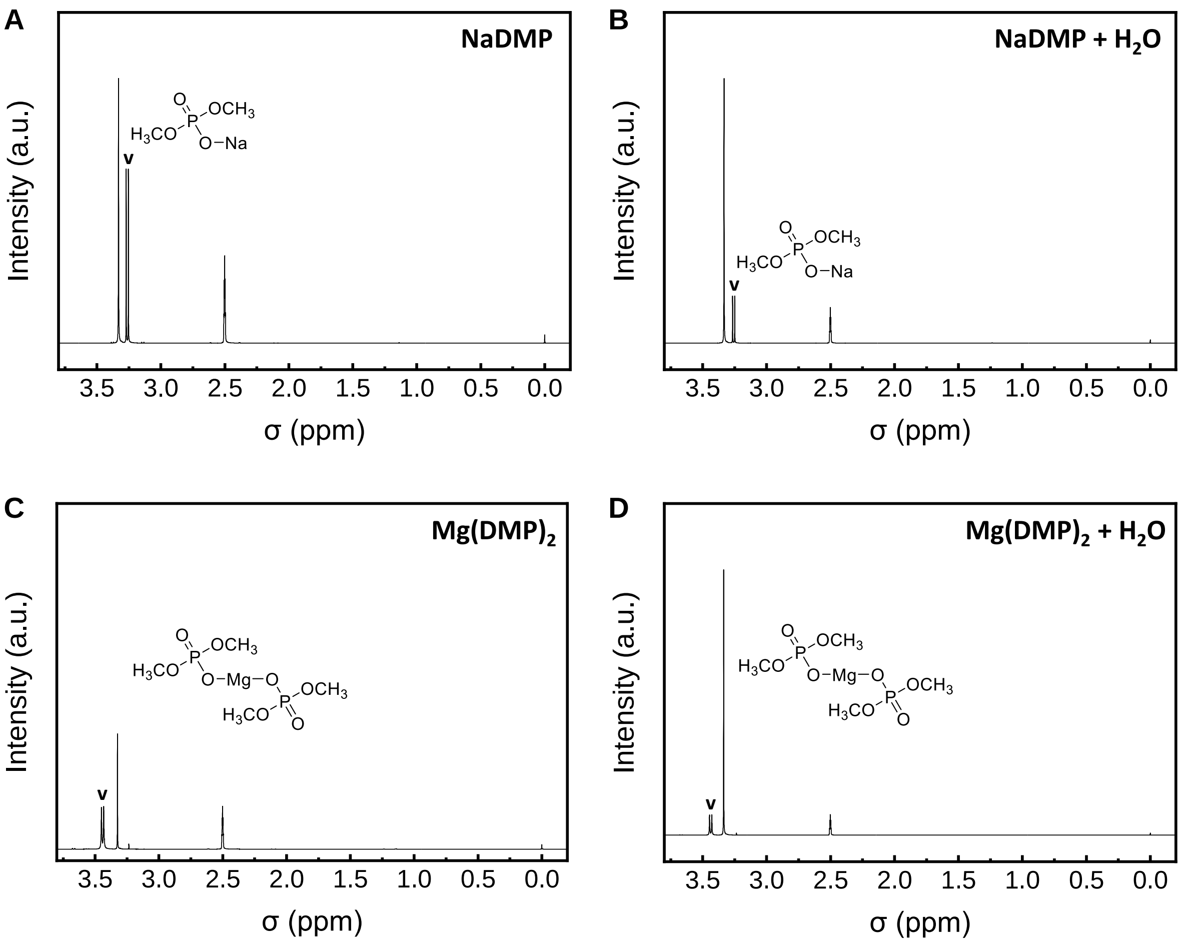

Supplementary Fig. 22. ^{1}H Nuclear magnetic resonance (NMR) spectra for (A) sodium dimethyl phosphate (NaDMP, synthesized),[2] and (B) NaDMP in the water. ^{1}H NMR spectra for (C) synthesized $Mg(DMP)_2$, and (D) reaction mixture of $Mg(DMP)_2$ and water.

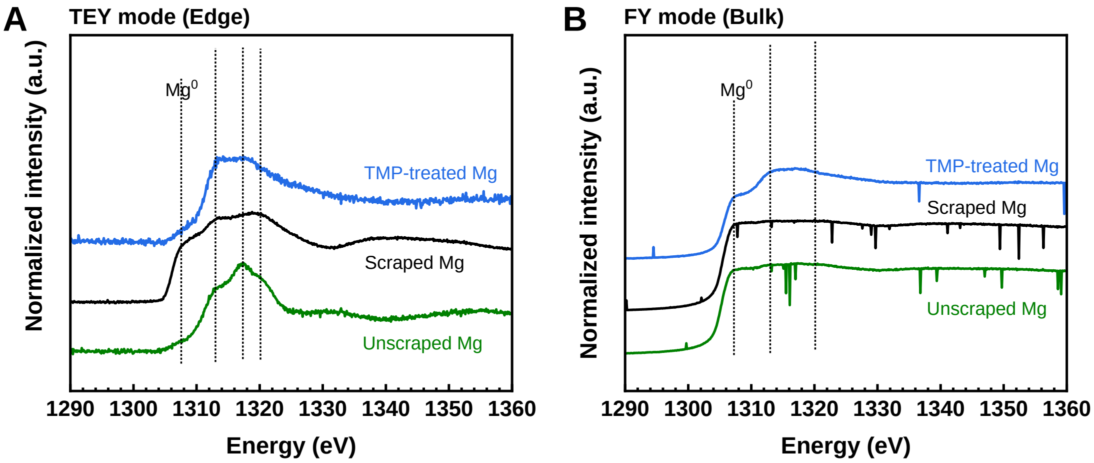

Supplementary Fig. 23. Mg K-edge X-ray absorption spectra of the trimethyl phosphate (TMP)-treated Mg electrode, scraped untreated Mg, and non-scraped Mg measured in (A) total electron yield (TEY) mode and (B) fluorescence yield (FY) mode, which provide the information on the electronic structure sensitive to surface region (~10 nm) and bulk (~100 nm), respectively.[3] Comparison between spectra measured in TEY and FY modes indicates that the surface region of TMP-treated Mg electrode is covered with thin layer whose nature is different from passive film formed on scraped or non-scraped Mg metals.

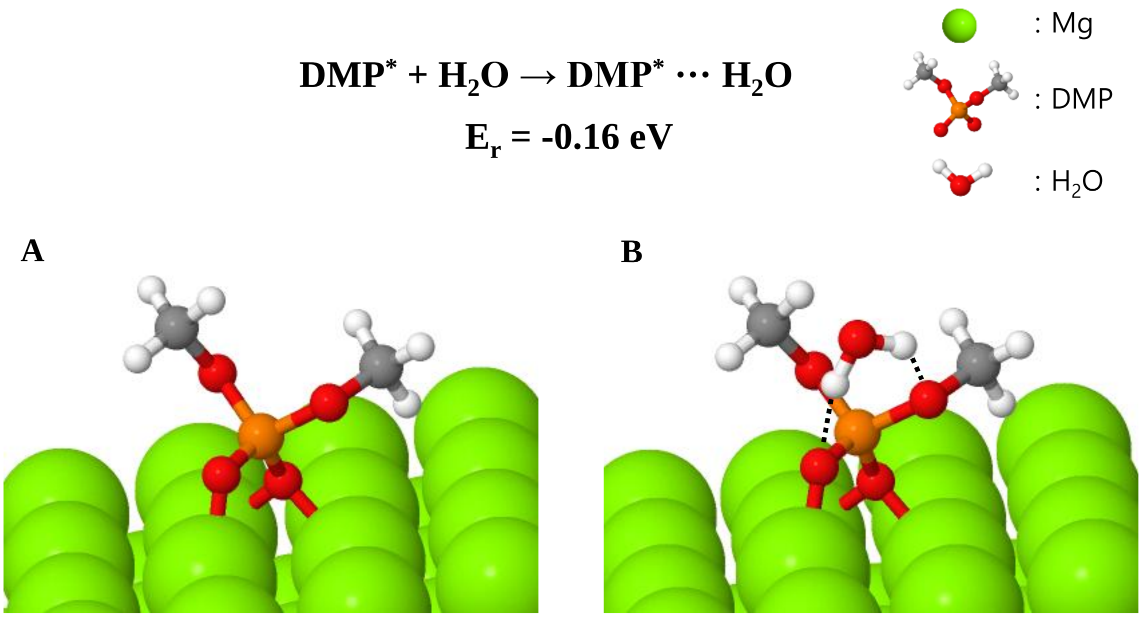

Supplementary Fig. 24. DFT calculation for (A) dimethyl phosphate (DMP) molecule adsorbed on a magnesium surface (DMP^*), and (B) the formation of a hydrogen bond between DMP and H_2O ($DMP^*\cdots H_2O$). The reaction energy for the hydrogen bonding interaction between the water molecule and DMP is –0.16 eV, indicating an exothermic process. This exothermic reaction is attributed to the hydrophilic nature of DMP as a protective film, which promotes hydrogen bonding with water molecules. Consequently, this interaction can inhibit the diffusion of H_2O to the Mg surface.

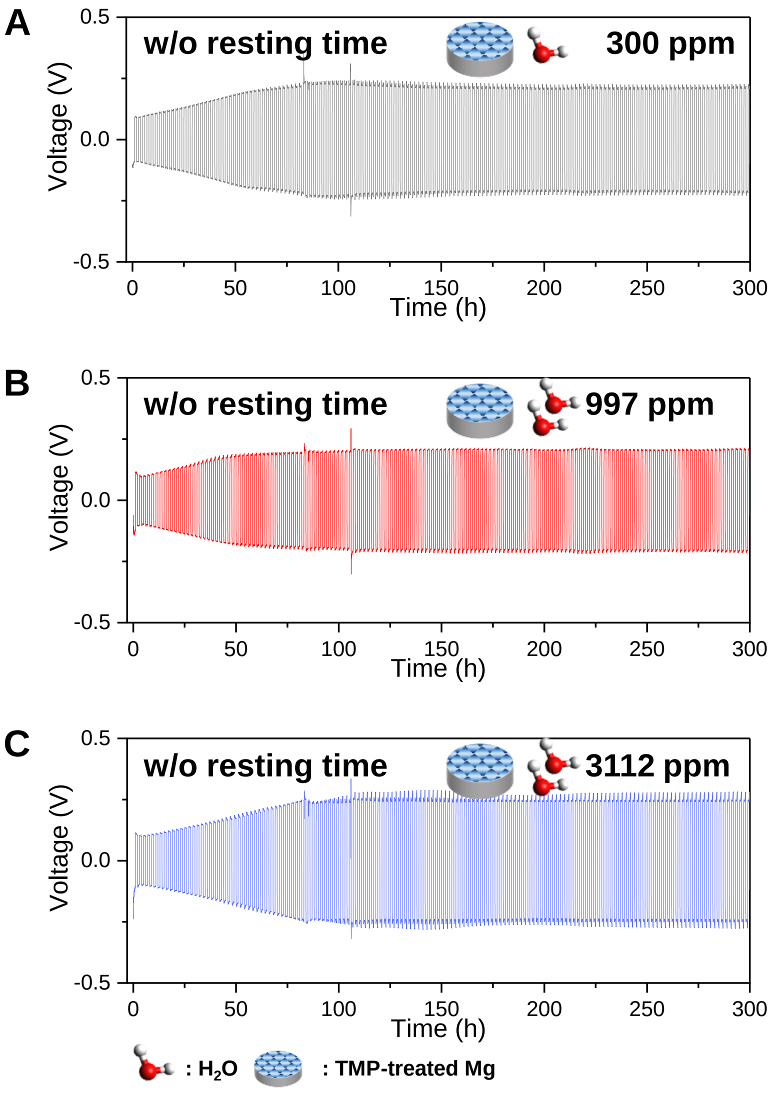

Supplementary Fig. 25. Mg plating-stripping behavior of symmetric cells using trimethyl phosphate (TMP)-treated Mg electrodes in E1 containing (A) 300 ppm, (B) 997 ppm, and (C) 3112 ppm of water content. All measurements were conducted without resting period after cell assembly. Stable Mg plating-stripping performance under this condition (no resting time) indicated that moisture-scavenging by Me_2Mg in the moist electrolyte was very fast and highly effective.

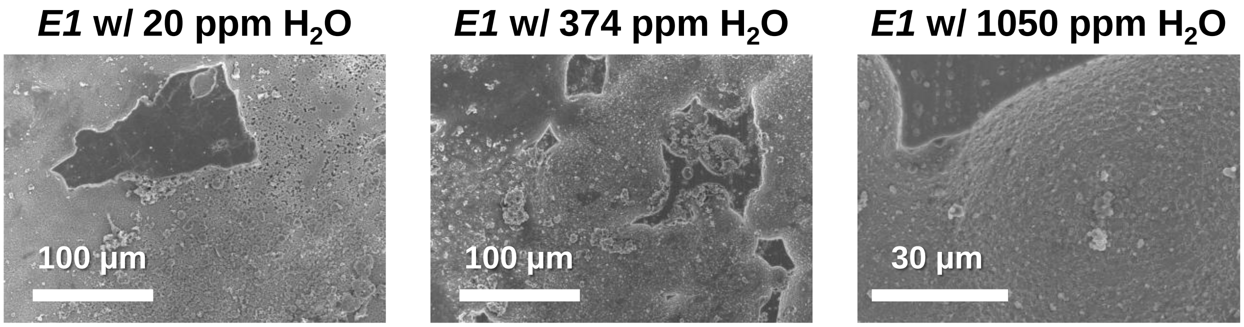

Supplementary Fig. 26. SEM images of trimethyl phosphate (TMP)-treated Mg electrodes after 10 plating-stripping cycles in symmetric cells using E1 electrolytes with 20 ppm, 374 ppm, and 1050 ppm of water. The dark regions indicate areas with limited reaction during TMP treatment, whereas the gray regions correspond to TMP-treated areas where smooth Mg deposition occurred, regardless of the initial moisture content in the electrolyte.

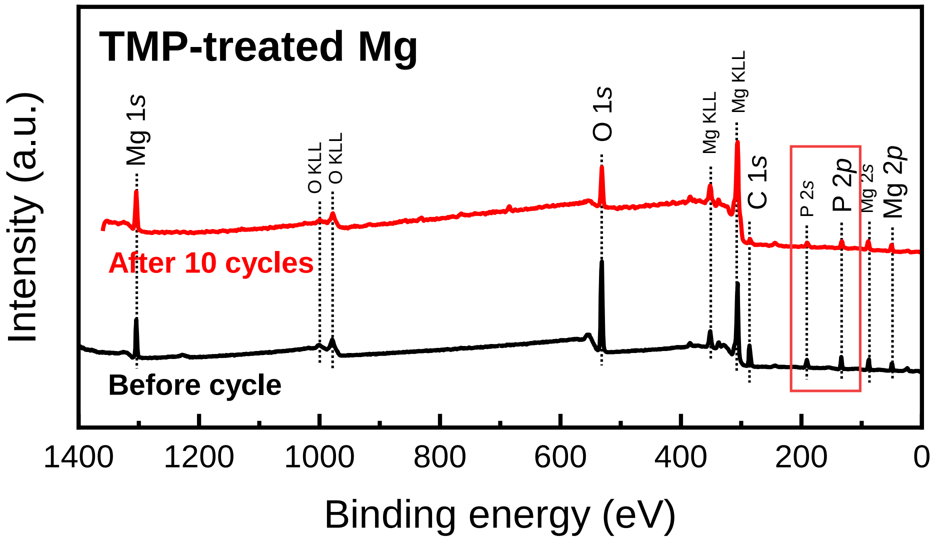

Supplementary Fig. 27. X-ray photoelectron spectroscopy (XPS) spectra (survey scan) of trimethyl phosphate (TMP)-treated Mg electrodes after 10 cycles of Mg plating-stripping in symmetric cells with E1 electrolytes containing 1050 ppm of water. The presence of the P 2*p* peak in cycled TMP-treated electrode indicated that the $Mg(DMP)_2$ protective layer was maintained after cycling.

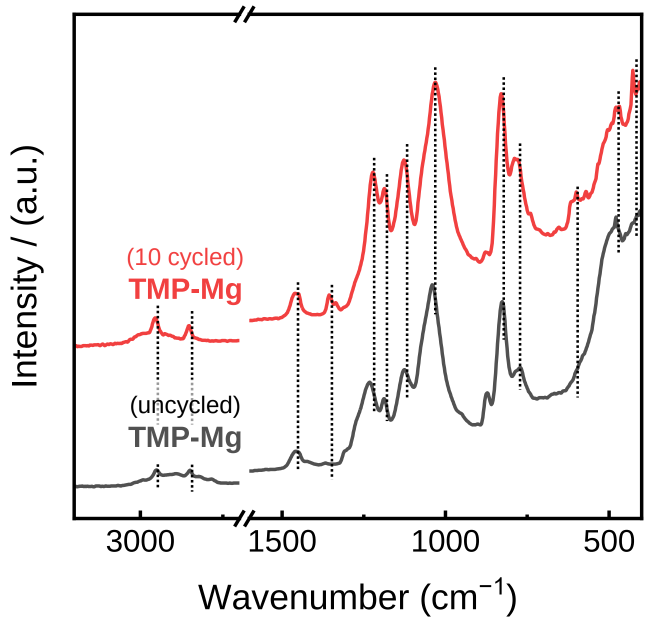

Supplementary Fig. 28. Fourier transform–infrared (FT-IR) spectra of trimethyl phosphate (TMP)-treated Mg electrodes before and after 10 cycles of Mg plating-stripping in symmetric cells. The spectra for $Mg(DMP)_2$ was clearly observed in the cycled TMP-treated electrode, indicating that $Mg(DMP)_2$-based protective layer was retained after cycling.

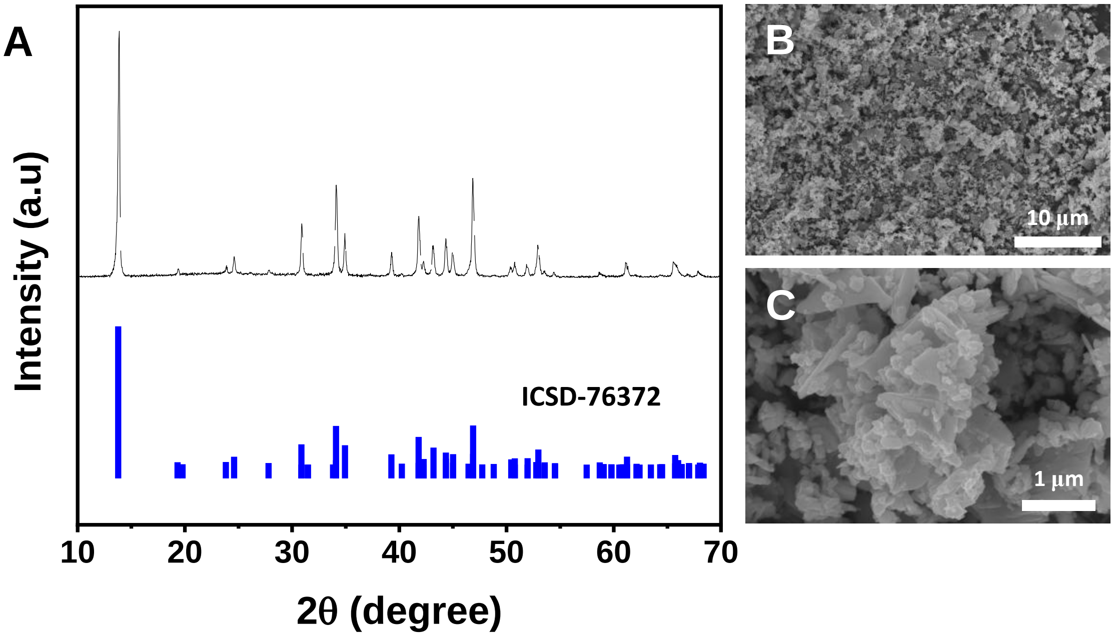

Supplementary Fig. 29. (A) X-ray diffraction pattern of Mo_6S_8 Chevrel phase with a relevant stick pattern (ICSD-76372), and (B), (C) its microstructure used in this work. Chevrel phase was synthesized by following molten salt method faithfully from the literature.[4, 5]

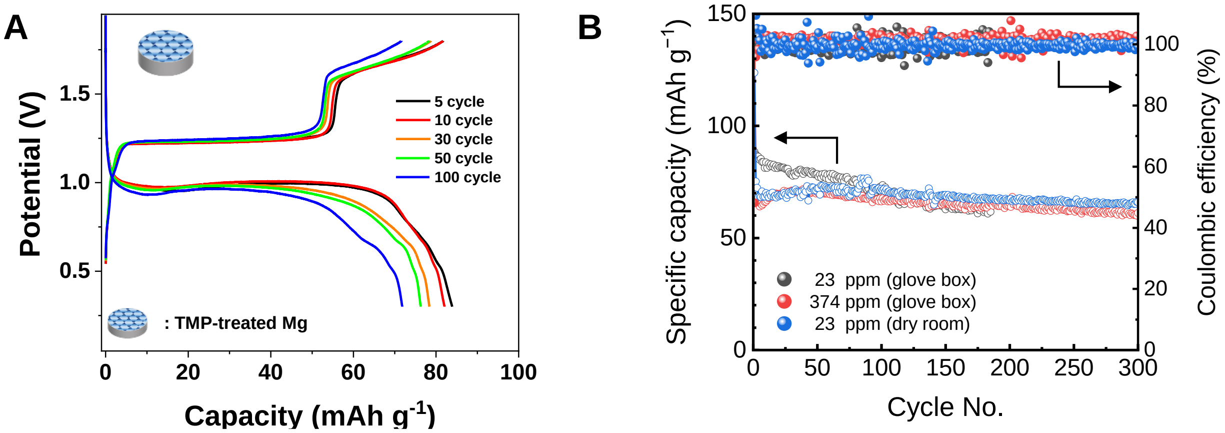

Supplementary Fig. 30. (A) The discharge-charge profiles of a full cell made of Mo_6S_8 Chevrel phase positive electrode and trimethyl phosphate (TMP)-treated Mg metal negative electrode in dry E1 electrolyte (23 ppm H_2O). The cell was assembled in an argon-filled glovebox. The current rate was 0.1C (1C = 128 mA g^{-1}). (B) The cyclic performance and corresponding Coulombic efficiencies of Mg||Mo_6S_8 full cells assembled (black) in a glovebox with dry E1 electrolyte (23 ppm), (red) in a glovebox with moist E1 electrolyte (374 ppm), (blue) in a dry-room facility with dry E1 electrolyte (23 ppm)

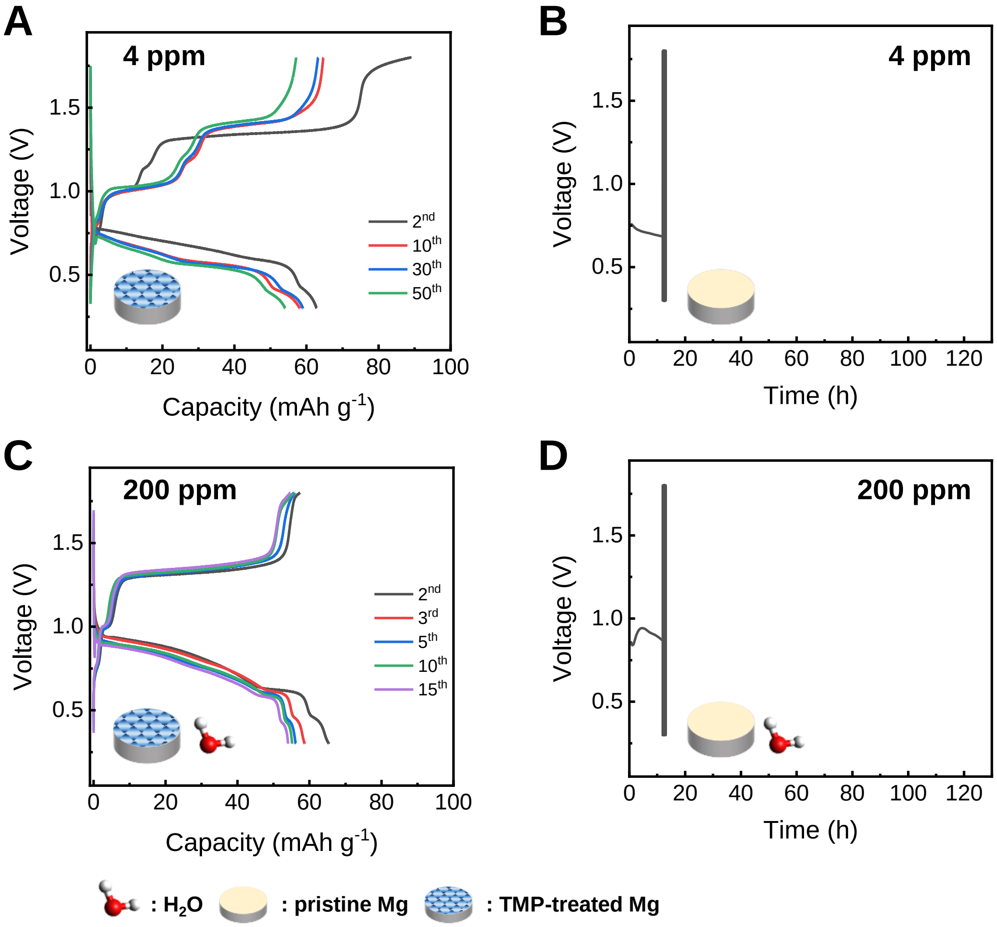

Supplementary Fig. 31. The discharge-charge profiles of the cells composed of Mo_6S_8 Chevrel phase positive electrodes and trimethyl phosphate (TMP)-treated or scraped Mg metal negative electrodes in dry (4 ppm H_2O) or moist (200 ppm) E3 electrolyte: (A) TMP-treated Mg metal in dry E3; (B) scraped Mg metal in dry E3; (C) TMP-treated Mg metal in moist E3; (D) scraped Mg metal in moist E3. The cells were assembled in an argon-filled glovebox. The current rate was 0.05C (1C = 128 mA g^{-1}).

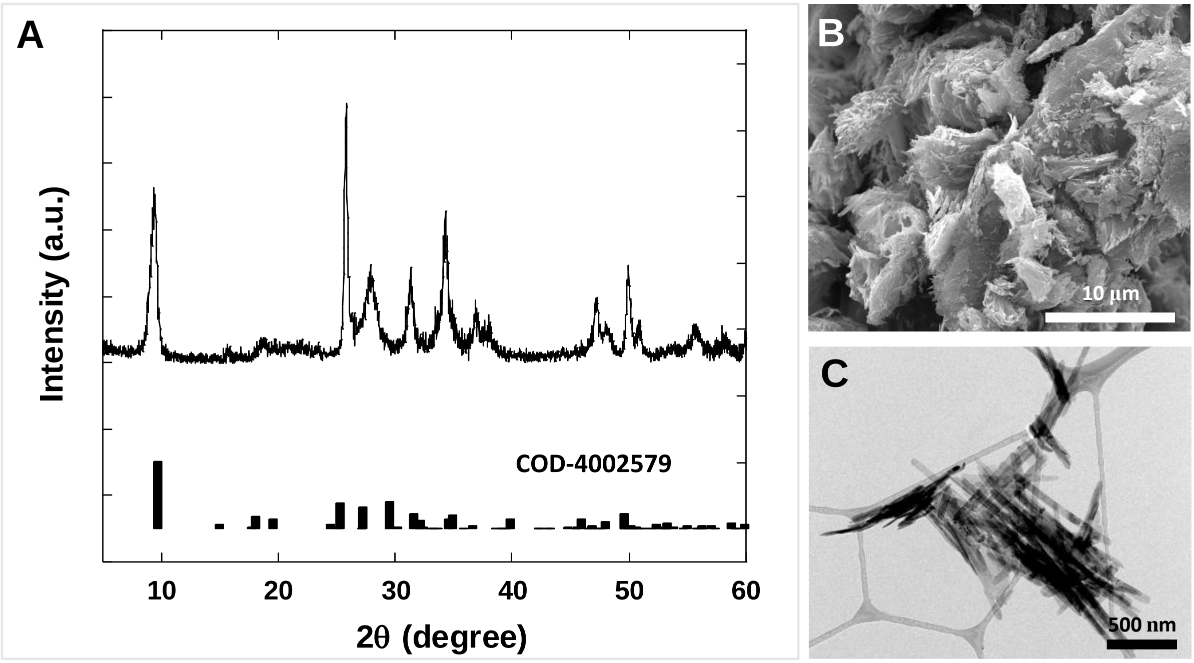

Supplementary Fig. 32. (A) X-ray diffraction pattern of $NH_4V_4O_{10}$ nanorods with a relevant stick pattern (COD-4002579), and (B), (C) its microstructure observed by (B) scanning electron microscopy (SEM) and (C) transmission electron microscopy (TEM).

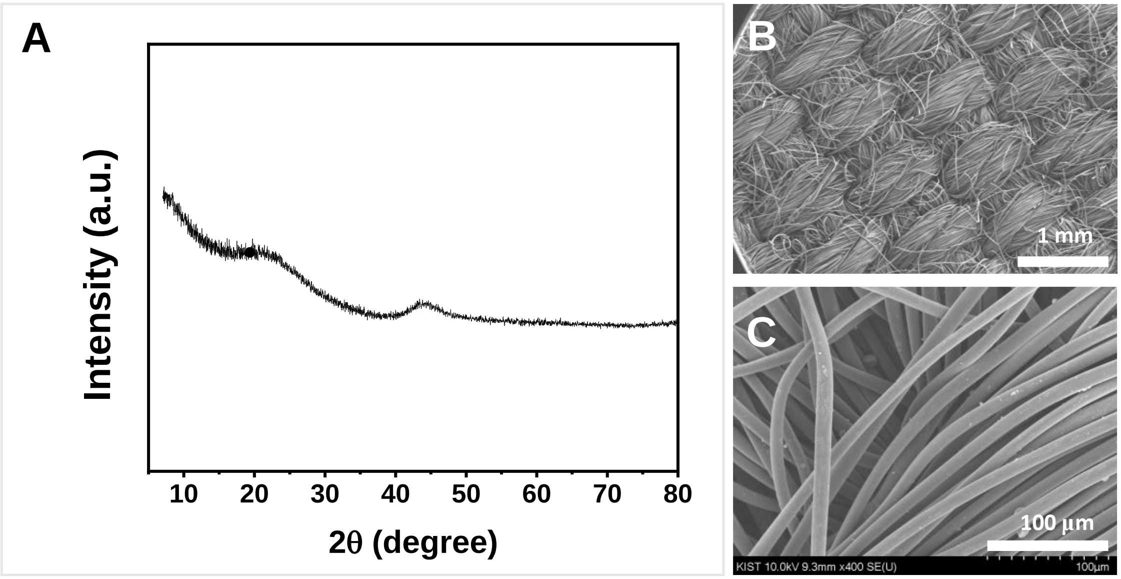

Supplementary Fig. 33. (A) X-ray diffraction pattern of activated carbon cloth (ACC) and (B), (C) its microstructure used in this work. ACC is poorly crystalline with a high surface area > 1,800 m^2 g^{-1}.

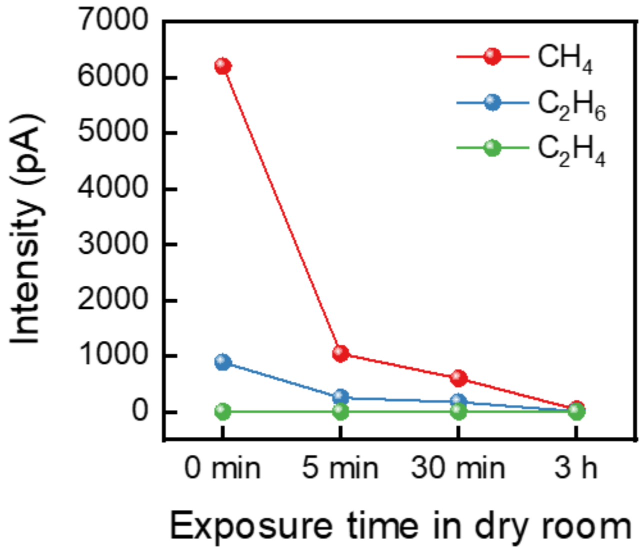

Supplementary Fig. 34. Amounts of methane, ethane, and ethylene gas measured by gas chromatography–flame ionization detector (GC-FID) after addition of moist E1 electrolyte (997 ppm H_2O) to trimethyl phosphate (TMP)-treated Mg metal disks exposed to a dry-room air for various period of time (0, 5, 30 min, and 3 h).

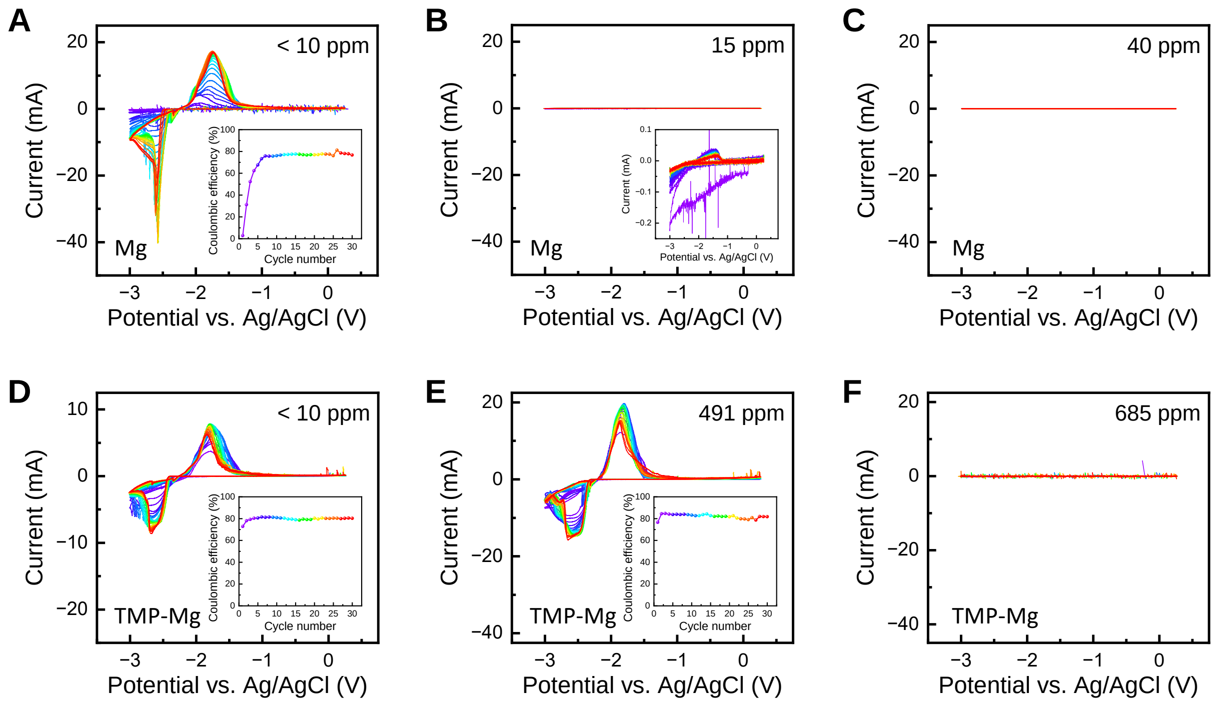

Supplementary Fig. 35. Cyclic voltammetry (CV) curves using the three-electrode electrochemical cells. The measurements were conducted with a stainless steel foil working electrode (WE). The reference electrode (RE) was Ag/AgCl immersed in dried E1 electrolyte. (A-C) CV profiles obtained using a scraped Mg counter electrode in E1 electrolyte containing various water contents. (D-F) CV profiles obtained using a trimethyl phosphate (TMP)-treated Mg counter electrode in E1 electrolyte containing various water contents. The inset in (A, D, E) show the corresponding Coulombic efficiency of the systems.

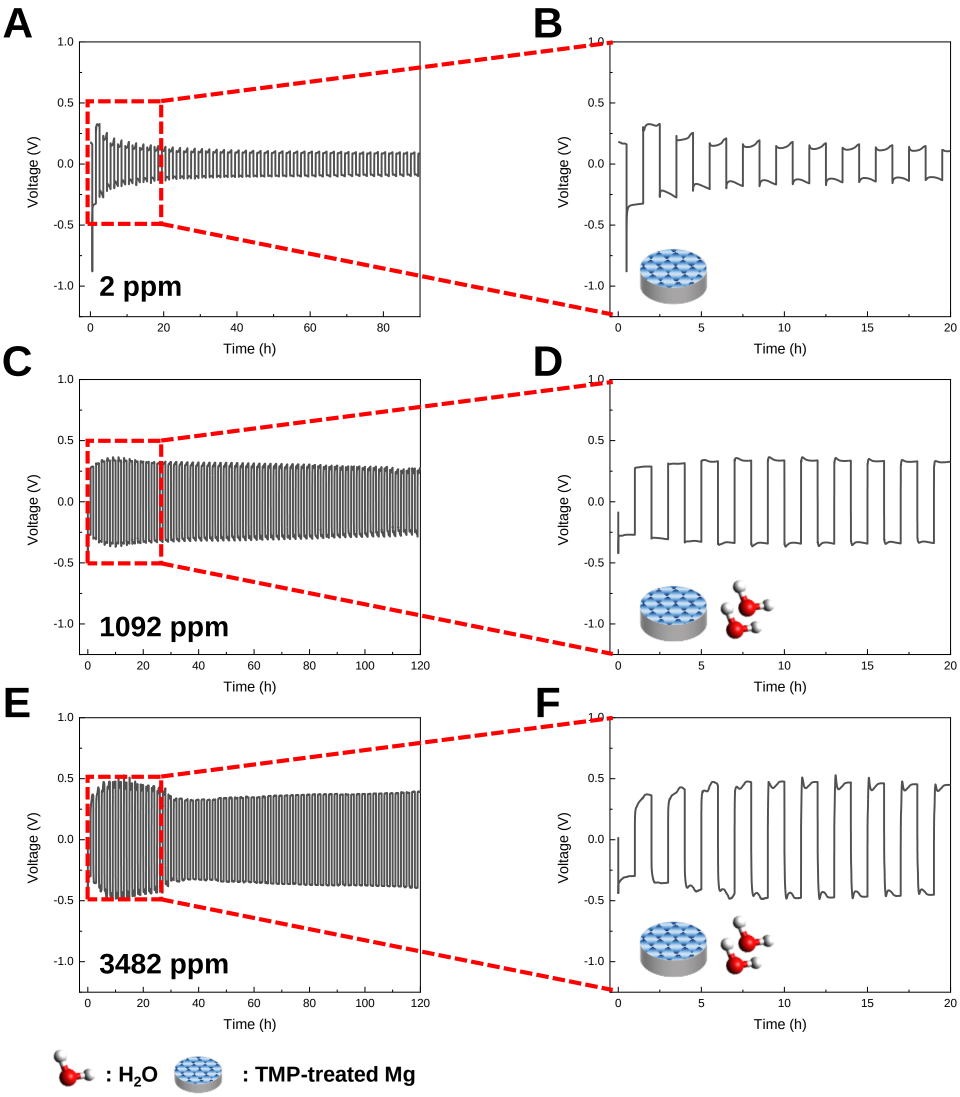

Supplementary Fig. 36. Mg plating-stripping performance of symmetric cells with trimethyl phosphate (TMP)-treated Mg electrodes in Mg[B(hfip)$_4$]$_2$-based E4 electrolyte (hfip = 1,1,1,3,3,3-hexafluoroisopropyloxy) containing different water contents. (A, C, E) Long-term cycling behavior using E4 electrolytes containing 2 ppm, 1092 ppm, and 3482 ppm of water, respectively. (B, D, F) Enlarged views of the first 20 cycles from (A), (C), and (E), respectively. The plating-stripping was carried out in 0.1 mA cm^{-2}, 0.1 mAh cm^{-2}.

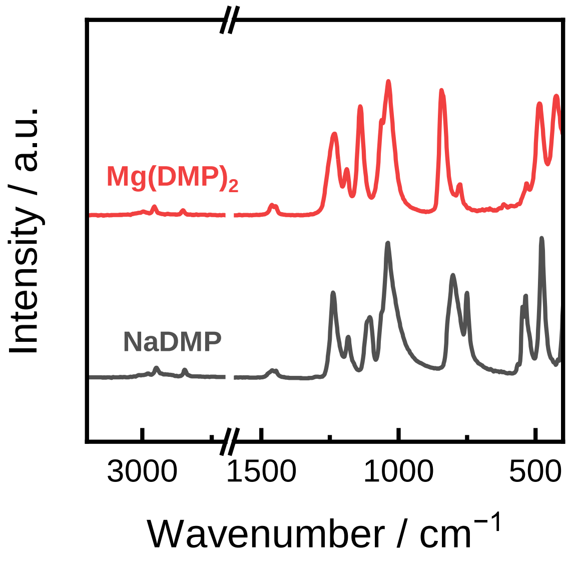

Supplementary Fig. 37. Fourier transform–infrared (FT-IR) spectra of synthesized magnesium dimethyl phosphate ($Mg(DMP)_2$) and NaDMP.

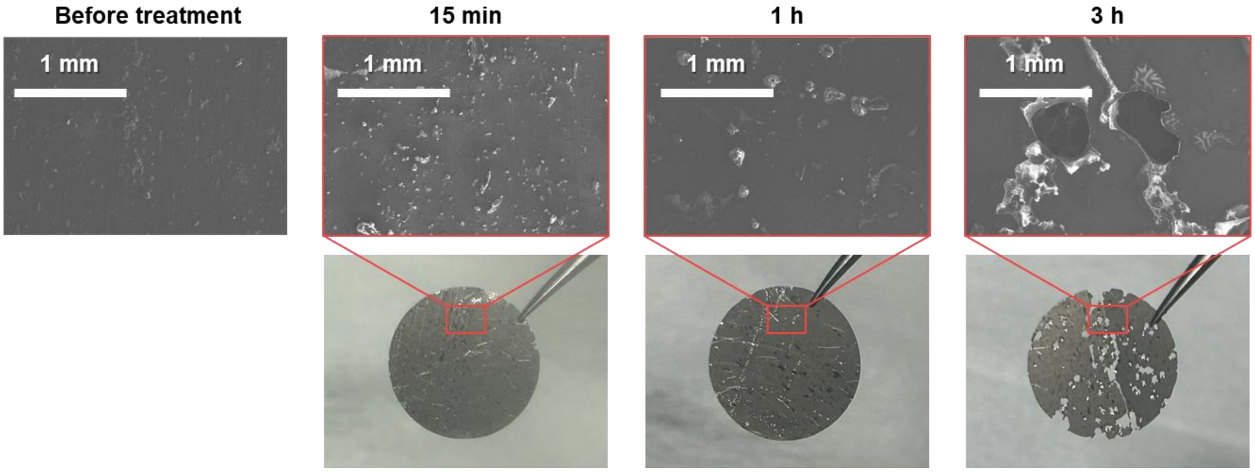

Supplementary Fig. 38. Scanning electron microscopy (SEM) images and photographs of non-scraped Mg metal foil disks before and after trimethyl phosphate (TMP) treatment (15 min, 1 h, and 3 h). For non-scraped Mg metal, reaction with TMP resulted in the development of reaction pits. The gradual increase in number and size of reaction pits was observed over time. After 1 hour of reaction time, the pit size grew up to ~100 μm. But, after 3 h of reaction time, the reaction seemed to too excessive as many large bored-through pits (~500 μm in diameter) were observed.

Supplementary Table 1. Mg content suspended in the solvent measured by inductive coupled plasma-optical emission spectrometer (ICP-OES) after 5 pieces of trimethyl phosphate (TMP)-treated or scraped Mg metal disks were immersed in 5 mL of moist G1 with 3135 ppm H_2O for 1 day.

**Mg metal in moist G1 (3135 ppm H_2O)**	**Mg content /** ppm
Scraped Mg	1.33
TMP-treated Mg	65.1

Supplementary Table 2. Residual H_2O content in 0.5 mL of electrolyte measured by Karl-Fisher titration tools after immersing 5 pieces of trimethyl phosphate (TMP)-treated Mg metal disks for 3 h.

**Initial H_2O content** / ppm	**Final H_2O content** / ppm	**Amount of H_2O scavenged** / ppm
3734.9	1622.4	2112.5
6606.4	3774.4	2832.0

Supplementary Table 3. Measurement of residual H_2O content in 0.5 mL of moist electrolyte after addition of 0.413 g magnesium dimethyl phosphate ($Mg(DMP)_2$) powder for 3 h by Karl-Fischer titration.

**Initial H_2O content / ppm**	**Final H_2O / ppm**	**Amount of H_2O scavenged / ppm**
3491.3	2828.4	662.9

To simulate the asymmetric coin-cell condition, where 0.1 mL of electrolyte was used per TMP-treated Mg disk, 0.413 g of $Mg(DMP)_2$ (the estimated amount corresponding to five TMP-treated Mg disks) was added to 0.5 mL of moist G1 solvent for this experiment.

Supplementary Table 4. Analysis on the Mg content dissolved in trimethyl phosphate (TMP) solution after extra amount of MgO or $Mg(OH)_2$ powder was added to the solution. The concentration was measured by inductive coupled plasma-optical emission spectrometer (ICP-OES).

**Mg species in TMP**	**Mg content** / ppm
Saturated MgO	42
Saturated $Mg(OH)_2$	40

Supplementary References

1. R. Salinger, H. Mosher, Infrared Spectral Studies of Grignard Solutions. *J. Am. Chem. Soc.* **86**, 1782-1786 (1964)

2. Hikawa H, Imani M, Suzuki H, Yokoyama Y, Azumaya I. Benzoyl methyl phosphates as efficient reagents in the one-pot tandem approach for the synthesis of 2-phenylbenzimidazoles in water. *RSC advances* **4**, 3768-3773 (2014).

3. Singh JP, Kim SH, Won SO, Lee I-J, Chae KH. Atomic-scale investigation of MgO growth on fused quartz using angle-dependent NEXAFS measurements. *RSC advances* **8**, 31275-31286 (2018).

4. Lancry E, Levi E, Mitelman A, Malovany S, Aurbach D. Molten salt synthesis (MSS) of $Cu_2Mo_6S_8$—New way for large-scale production of Chevrel phases. *Journal of Solid State Chemistry* **179**, 1879-1882 (2006).

5. Lee B, *et al.* Investigation on the structural evolutions during the insertion of aluminum ions into Mo_6S_8 Chevrel phase. *Journal of the Electrochemical Society* **163**, A1070 (2016).
