# Supplementary material for: Moisture-tolerant Mg-metal electrodes for practical fabrication of rechargeable Mg batteries
Source: Nat Commun. 2026 Mar 9;17:3678. doi: 10.1038/s41467-026-70378-3 (PMC13100027; doi:10.1038/s41467-026-70378-3)
Supplement: Supplementary file 2 — Description of Additional Supplementary Files [file 41467_2026_70378_MOESM2_ESM.docx]

**Description of Additional Supplementary Files**

File name: Supplementary Data 1

Description:

This supplementary data file provides the atomic coordinates of the optimized structures obtained from DFT calculations associated with Supplementary Fig. 24.

Mg.txt

Atomic coordinates of the optimized pristine Mg slab model.

Mg-DMP.txt

Atomic coordinates of the optimized Mg slab with an adsorbed DMP molecule.

Mg-DMP-H2O.txt

Atomic coordinates of the optimized Mg slab with a DMP molecule and a co-adsorbed H_2_O molecule
